# Genetic determinants of proteomic aging

**DOI:** 10.1038/s41514-025-00205-4

**Published:** 2025-04-26

**Authors:** Alexander Mörseburg, Yajie Zhao, Katherine A. Kentistou, John R. B. Perry, Ken K. Ong, Felix R. Day

**Affiliations:** 1 MRC Epidemiology Unit, Institute of Metabolic Science, University of Cambridge, Cambridge, UK; 2 Metabolic Research Laboratories, Institute of Metabolic Science, University of Cambridge, Cambridge, UK; 3 Department of Paediatrics, University of Cambridge, Cambridge, UK

**Keywords:** Ageing, Genome

## Abstract

Changes in the proteome and its dysregulation have long been known to be a hallmark of aging. We derived a proteomic aging trait using data on 1459 plasma proteins from 44,435 UK Biobank individuals measured using an antibody-based assay. This metric is strongly associated with four age-related disease outcomes, even after adjusting for chronological age. Survival analysis showed that one-year older proteomic age, relative to chronological age, increases all-cause mortality hazard by 13 percent. We performed a genome-wide association analysis of proteomic age acceleration (proteomic aging trait minus chronological age) to identify its biological determinants. Proteomic age acceleration showed modest genetic correlations with four epigenetic clocks (*R*_g_ = 0.17 to 0.19) and telomere length (*R*_g_ = −0.2). Once we removed associations that were explained by a single pQTL, we were left with three signals mapping to *BRCA1*, *POLR2A* and *TET2* with apparent widespread effects on plasma proteomic aging. Genetic variation at these three loci has been shown to affect other omics-related aging measures. Mendelian randomisation analyses showed causal effects of higher BMI and type 2 diabetes on faster proteomic age acceleration. This supports the idea that obesity and other features of metabolic syndrome have an adverse effect on the processes of human aging.

## Introduction

Health declines as humans age, and age is the most important risk factor for most non-communicable diseases^1^. By elucidating the biology that changes as people age, we may be able to identify specific pathways that increase the risk of disease. This is especially pertinent given the global trend toward an aging population, the successes in treating communicable diseases, and the shift in the global disease burden toward non-communicable diseases^2^.

Chronological age is an imperfect proxy for the physiological changes that occur during aging. Researchers have attempted to capture the biological basis of aging using omics data over the last decade, generating so-called ‘aging-clocks’. Pioneering studies by Hannum et al.^3^ and Horvath (2013)^4^ used cytosine methylation patterns at CpG dinucleotides, an important epigenetic marker. These are highly accurate predictors of chronological age and show a high degree of reproducibility^5,6^. In most types of cancers, tumour tissues show methylation patterns associated with older ages compared to matched tissue from the same individual^4,7^. It is assumed that individuals whose methylation predicted age is very different to their chronological age are aging biologically at different rates^3^. These clocks highlighted an impact of the enzyme TERT^5^, immune cell traits, and showed mixed evidence regarding the role of blood lipid levels on the rate of epigenetic aging^6,8^.

Studies have also explored genetic influences on these clocks. Gibson et al.^9^ identified ten loci associated with Horvath’s epigenetic age acceleration (EAA) and one with Hannum EEA in 13,493 European ancestry individuals. A more comprehensive meta-analysis of 40,950 individuals of African American and European ancestry found 137 genome-wide significant loci across six different methylation-based biomarkers^10^. However, no distinct clusters or pathways have been conclusively identified from the genes underlying epigenetic clocks. McCartney et al.^10^ demonstrated that a polygenic score for GrimAge, which is trained on mortality risk including epigenetic markers of smoking, was strongly associated with both adiposity-related traits and parental longevity. This link was confirmed by Mendelian randomisation analyses showing that increased adiposity was causally related to accelerated aging. Other Mendelian randomisation studies have shown that smoking and alcohol intake are associated with faster aging^11^, some evidence of links to the immune response^6^, and inconclusive results about the links to cancer^12^.

While these epigenetic studies have been informative in understanding the biological mechanisms of aging, it is unclear whether the findings are specific methylation-measured aging. Plasma proteins might represent a more biologically interpretable signal compared to DNA methylation marks^13^, thus investigating biological aging using proteomics might lead to more actionable insights. The first plasma protein-based aging clock was described by Tanaka et al.^14^, in a small cohort (*N* = 240) typed for 1301 proteins. They highlighted GDF15 as having the strongest association with age and suggested that immune and neuronal pathways were enriched among the proteins that showed a significant age-association. Lehallier et al.^15^ built an improved proteomic clock consisting of 373 proteins derived from 4263 participants. The authors were able to demonstrate that individuals who exhibited higher proteomic aging performed worse on cognitive and physical tests. Two recent studies developed proteomic aging clocks based on plasma protein data from UK Biobank participants, with total sample sizes of 45,441 and 53,021, respectively^16,17^. These clocks accurately predict all-cause mortality, multimorbidity and the onset of several age-related diseases. Here we seek to explore the genetic underpinnings that are related to proteomic aging.

We sought to extend this literature by assessing proteomic aging estimated using 1459 circulating proteins in 44,435 individuals from the UK Biobank study, identifying a number of novel phenotypic and genetic determinants. In addition, by focusing on the genetic effects that underpin these changes, we can extend our model to answer question about the determinants of accelerated aging, potentially identifying the causes of very general effects on morbidity.

## Results

### Estimation of a proteomic aging trait

**Fig. 1:**
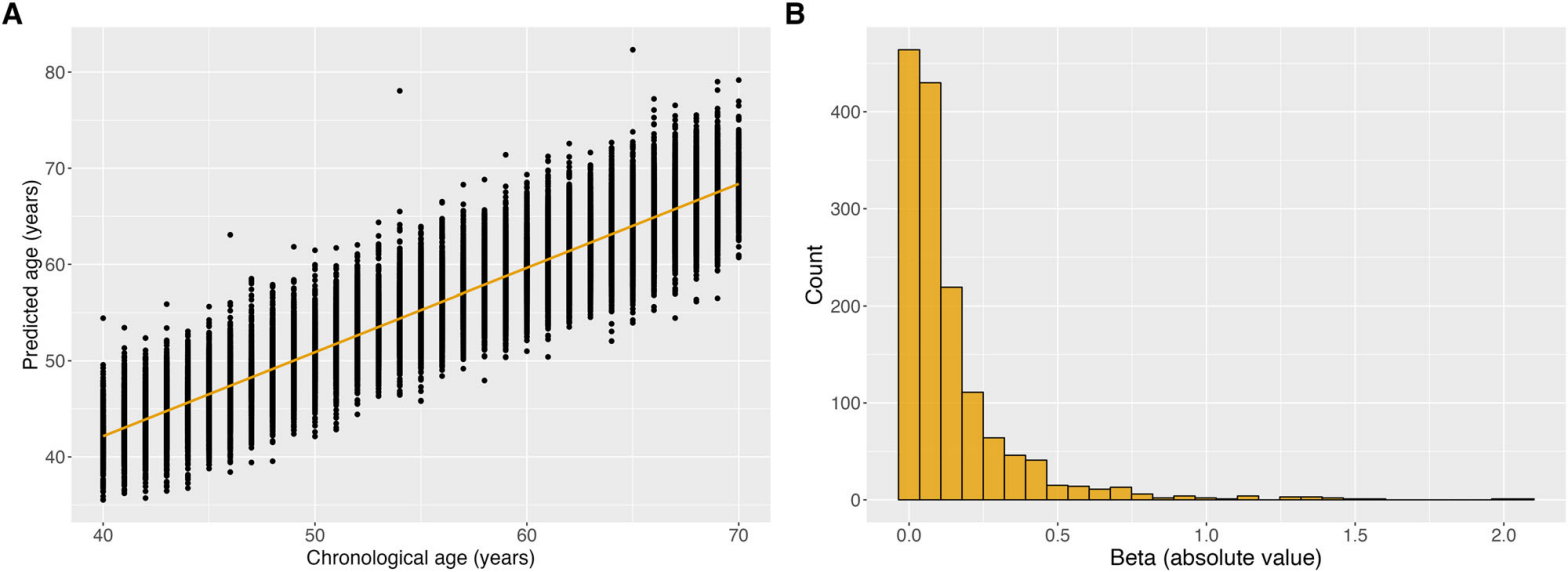
Performance and feature contributions of the aging clock model. **A** Performance of the elastic net model for chronological age trained on Olink data in the UK Biobank cohort (*N* = 44,435). **B** Distribution of elastic net model coefficients for individual Olink proteins (*N* = 1459).

To identify plasma proteins that change with chronological age in adulthood, we assessed 1459 circulating proteins in 44,435 people in the UK Biobank study, measured using the antibody-based Olink Explore 1536 assay. We first ran an elastic net model to train a predictor of chronological age (Supplementary Table 1). Predicted proteomic age was, as expected, highly correlated with reported age: Pearson’s *R* = 0.94, mean absolute error (MAE) 2.3 years (Fig. 1). In general, aging was associated with a greater abundance of proteins when considering the effect direction from univariate models (Supplementary Table 1). We computed SHapley Additive exPlanations (SHAP) values, a robust framework to assess feature importance^18^, to detect the top 20 proteins in our model (Supplementary Table 2). Many of these top 20 proteins have been previously implicated in age-related diseases. For example, GDF15 is a stress-regulated hormone which decreases food intake and is hypothesized to contribute to frailty^19^. The role of EGFR, a receptor tyrosine kinase, in cancer is also well-studied^20^. ITGAV, a member of the integrin family of extracellular matrix proteins, has been shown to influence tumour progression and tissue invasion across various cancer types^21^. Given the well-established links between measures of aging and cancer, we identified the intersection of these top proteins with the results of a recent study of protein-cancer associations in UK Biobank^22^ (Supplementary Table 3). Ten of the twenty showed an association with one or more cancers, suggesting that the proteomic model is identifying proteins related to disease-relevant aging.

Intriguingly, the top-ranked protein SCARF2 is a predictive biomarker of liver cancer. While most of these ten associations were directionally consistent (proteins upregulated in aging were also elevated leading up to a cancer), this was not always the case. The exceptions were THBS2, SPON2 and ROBO1. That these markers appear upregulated in cancer but downregulated in aging, may be due to the way that elastic net models partition variance, but we do not rule out the presence of complex feedback loops in these pathways.

### Phenotypic associations of proteomic aging with disease outcomes

We investigated proteomic aging associations with selected age-related diseases, and other age-related traits. Predicted proteomic age was strongly associated with all the selected outcomes (Table 1). Indeed, predicted proteomic age completely attenuated the effect of chronological age on four high-level disease outcomes (Supplementary Table 4). This indicates that the proteome may be a major mediator of human aging on major disease outcomes. We also saw associations independent of chronological age with two continuous aging outcomes (LOY and age at menopause). However, the effects of chronological age were only modestly attenuated. Hence, predicted proteomic age appears to capture a component of biological aging with broad relevance to health.

**Table 1:** Protein age (estimated in years) associations with selected age-related diseases and continuous traits

Binary Outcomes	Prot. Age OR	Prot. Age 95% CI	Prot. Age *P*	Chron. Age OR	Chron. 95% CI	Chron. Age *P*
Cancer *(any)*	1.029	[1.021–1.038]	9.9×10^–12^	1.043	[0.997–1.091]	0.07
Cancer *(minus blood cancers)*	1.024	[1.015–1.033]	2.8×10^–8^	1.059	[1.011–1.109]	0.015
Type 2 Diabetes	1.082	[1.069–1.096]	9.1×10^–35^	1.032	[0.960–1.110]	0.388
Ischemic Heart Disease	1.084	[1.073–1.095]	2.3×10^–55^	1.044	[0.982–1.109]	0.167

Continuous Outcomes	Prot. Age Beta	Prot. Age SE	Prot. Age *P*	Chron. Age Beta	Chron. Age SE	Chron. Age *P*
Loss of the Y Chr. [scaled to max 2]	0.005	0.001	2.8×10^–4^	−0.071	0.006	5.5×10^–29^
Age at menopause [years]	−0.127	0.017	1.9×10^–14^	1.599	0.102	2.0×10^–54^

Models include proteomic age, chronological age, chronological age^2^ and where appropriate sex.

**Fig. 2:**
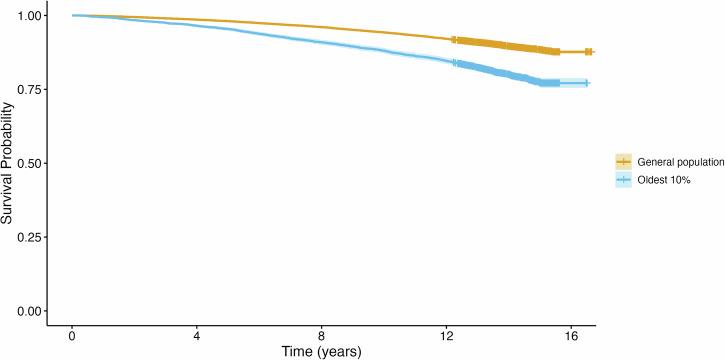
Proteomic aging stratifies individuals into divergent mortality trajectories. Kaplan-Meier plot showing the relationship between predicted proteomic age and all-cause mortality. Time (years) since study baseline assessment visit. Oldest 10% (blue line) indicates the oldest decile of proteomic age (adjusted for chronological age; *P* = 1.61×10^–120^). 95% confidence intervals are shown in lighter shading.

In a Cox proportional hazards model, each year older predicted proteomic age was associated with earlier all-cause mortality (hazard ratio=1.13, 95%CI = 1.12–1.14). To illustrate this association, we plotted survival in the 10% of UK Biobank individuals with the oldest proteomic age (adjusted for reported age) compared to the rest of the population (Fig. 2).

### Genome-wide association signals with proteomic age acceleration

We calculated the difference between predicted proteomic-age and reported chronological age (termed “proteomic age acceleration”) as a proxy for an individual’s rate of biological aging^13^. To identify genetic signals that confer faster proteomic aging we ran a genome-wide association study (GWAS) on proteomic age acceleration using the UK Biobank data. We also ran GWAS studies on proteomic aging traits generated separately in UK Biobank men and women. The genetic correlation between the proteomic ages calculated in UK Biobank men and women was 0.82 (95%CI: 0.59 to 1.06, *P* = 7.6×10^–12^). A sex-specific replication analysis of the genome-wide significant loci supports the similarity in genetic architecture across sexes, though the number loci is small (Supplementary Fig. 2).

**Fig. 3:**
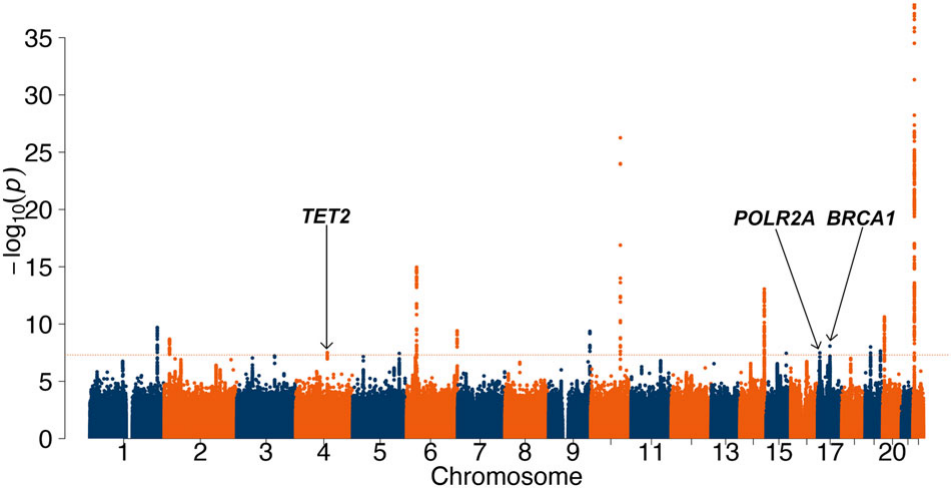
GWAS of proteomic age acceleration. Manhattan plot of GWAS results for proteomic age acceleration as outcome. The dashed orange line denotes the genome-wide significance threshold (*P* = 5×10^–8^).

In UK Biobank, we identified 16 independent GWAS signals (*P* < 5×10^–8^) with proteomic age acceleration (Fig. 3, Supplementary Table 5). To identify signals with widespread effects on plasma proteins and where the effect on accelerated aging was not driven by any specific pQTL, we identified all proteins associated with each of the 16 signals (pQTLs, at *P* < 3.4×10^–5^). Nine of the loci had more than one strongly associated protein. To investigate whether the links to age-acceleration were driven only by these proteins we then generated separate elastic net models for each signal, where we excluded all signal-associated proteins. Then we tested whether each signal was still associated with proteomic age acceleration. Some proteomic age acceleration signals appear to be wholly driven by a specific pQTL (Supplementary Table 5). For example, the signal at *SCARF2* (*P* = 1.3×10^–38^) is a strong cis-pQTL for SCARF2 (and is not associated with other proteins) which is the second most highly weighted protein in the elastic net model. This signal was no longer associated with a proteomic aging trait that explicitly excluded SCARF2 protein concentrations (*P* = 5.8×10^–3^).

Three signals appeared to have widespread effects on proteomic age acceleration, showing little attenuation in elastic net models that excluded their specific pQTLs. We used the “GWAS to genes” method to link signals to genes (Supplementary Table 6). Of our three genes of interest one was at strongly linked to both *TET2* and to *PPA2* – with PPA2 ranked 36^th^ and TET2 ranked 38^th;^ however, given the abundant literature linking *TET2* to other aging phenotypes we suggest that *TET2* is the likely causal gene. The *TET2* gene encodes a DNA demethylation enzyme and is mutated in clonal haematopoiesis of indeterminate potential (CHIP). The other two signals were at *BRCA1*, coding for a DNA repair protein and associated with increased breast cancer risk, and *POLR2A*, encoding a subunit of RNA polymerase II. In a recent survival analysis also using the UK Biobank whole exome sequencing data (in a much larger data set not limited by available proteomics) gene burden tests showed that protein-truncating variants (PTVs) in both *BRCA1* and *TET2* were associated with reduced individual and parental lifespan^23^. The association of pathogenic variants in *BRCA1* and shortened lifespan was independently replicated in a large study of Icelandic individuals^24^. We sought to validate our findings using GWAS data from four previously published epigenetic aging clocks (Supplementary Table 7): Hannum^3^, IEAA (intrinsic epigenetic age acceleration) which is a derivative of the Horvath clock adjusted for abundance measures of blood cell counts^25^, DNAm PhenoAge^26^ and GrimAge^27^. We detected positive genetic correlations with Hannum (*R*_*g*_ = 0.17, 95%CI: 0.03 to 0.32, *P* = 0.02), IEAA (*R*_*g*_ = 0.19, 95%CI: 0.06 to 0.33, *P* = 0.006), DNAm PhenoAge (*R*_*g*_ = 0.18, 95%CI: 0 to 0.36, *P* = 0.04) and DNAm GrimAge (*R*_*g*_ = 0.19, 95%CI: 0.03 to 0.36, *P* = 0.02). We observed negative genetic correlations of proteomic age acceleration with telomere length (*R*_*g*_ = −0.20, 95%CI: -0.28 to -0.13, *P* = 5×10^–8^) and age at natural menopause (*R*_*g*_ = -0.07, 95%CI: -0.14 to -0.01, *P* = 0.03) while there was no detectable relationship to breast cancer risk (*R*_*g*_ = -0.04, 95%CI: -0.11 to 0.03, *P* = 0.29).

A lookup of the three loci we identified - *BRCA1*, *TET2*, *POLR2A* (Supplementary Table 8) – found that the proteomic age acceleration increasing allele at *BRCA1* showed a strong negative association with telomere length (beta = -0.016, *P* = 6.2×10^–14^) and was also nominally associated with decreased IEAA. The proteomic age acceleration increasing allele at *TET2* was nominally associated with increased PhenoAge. This suggests that the effect of *TET2* on proteomic aging could be mediated by its effect on epigenetic aging. However, the *TET2* sentinel SNP rs9884482 was uncorrelated with the strongest signal (rs144317085) in the TET2 region for the IEAA clock (LD: *R*^2^ = 10^–4^, *P* = 0.81 in 1KG Europeans based on LD Pair Tool NCBI)^10^.

### Exploration of rare variant associations with proteomic aging

To identify rare variants associated with proteomic age acceleration we ran gene-based burden tests using different classes of rare putatively damaging variants (Supplementary Note, Supplementary Table 9**, Methods**). No genes passed the multiple-test corrected statistical significance threshold in our analysis. We did however observe a nominally significant negative association of PTVs in *TET2* and proteomic age acceleration (beta = -1.6, SE = 0.5, *P* = 5×10^–4^, carrier N = 36), supporting the previously highlighted common variant association. Due to the prominent role of *TET2* in CHIP we extracted possible somatic CHIP mutations. However, these were not associated with proteomic age acceleration (beta = -0.64, SE = 0.7, *P* = 0.4), indicating that the above association is driven by germline variation.

### Systematic look-up of signals associated with proteomic aging in other databases

The three ‘widespread-acting’ GWAS signals for proteomic age acceleration also showed associations with other phenotypes in the Open Targets and FinnGen databases (Supplementary Table 10). The variant 17:41351104_AT_A at *BRCA1* was absent in most public databases, so we used rs8070085, a proxy (*R*^2^ = 0.62 in imputed UK Biobank SNP array data).

The proteomic age acceleration increasing allele at *BRCA1* was associated with later age at menopause^28^ and with various blood fraction phenotypes, but not associated with breast cancer based on data from the Breast Cancer Association Consortium (BCAC) (Supplementary Table 8). The proteomic age acceleration increasing allele at *POLR2A* was associated with various traits, including decreased levels of sex-hormones (SHBG levels, total testosterone level), lower haematocrit and decreased risk for Leiomyoma of uterus. The proteomic age acceleration increasing allele at *TET2* showed associations with decreased total testosterone and total serum protein, as well as decreased breast cancer risk and various associations with white-blood cell related traits.

### Causal influences on accelerated proteomic aging

Previous work has shown that BMI and T2D have a very widespread effect on the proteome^29,30^, and we were interested to understand if there was a causal relationship between features of the metabolic syndrome and proteomic aging. We used Mendelian randomisation to assess the effect of BMI, type 2 diabetes and IGF1 on the outcome of proteomic age acceleration (Supplementary Table 11**)**. We observed significant effects of higher BMI and type 2 diabetes on faster proteomic aging. In the case of BMI, we saw evidence of significant pleiotropy, evidenced by the significant Egger’s intercept, but here the Egger’s MR *P*-value was also significant. As sex hormones have been suggested to play a role in sex-specific metabolic effects^31^ we were also interested to see if there was an impact of sex-hormone levels and found that higher levels of testosterone led to younger proteomic ages in women. Finally, there might have been a link between some phenotypes that represent genomic-aging, namely Loss-of-Y in men and age at menopause in women, both of which have been shown to broadly capture the body’s ability to maintain DNA integrity^28,32^. Here, there was a nominally significant effect of earlier menopause age, though interestingly this association (earlier menopause, older predicted proteomic age) was in a different direction to that seen with the specific SNP at the *BRCA1* locus.

## Discussion

This work extends our understanding of the processes of biological aging. Using normalised plasma protein measurements obtained with an antibody-based approach, we trained a chronological age predictor. We show that this measure of proteomic aging is associated with several key age-related diseases. This enabled us to identify genetic regions associated with general patterns of proteomic aging highlighting *TET2*, *BRCA1* and *POLR2A* as important drivers of this phenotype. Our results suggest that the relationships between older chronological age and common disease risks are potentially driven by protein dysregulation. Finally, we used the genetic data to perform Mendelian randomisation analyses that showed that higher BMI and type 2 diabetes appear to causally increase the rate of proteomic aging.

Some of the specific proteins that were highly weighted by the elastic net show consistency with those previously reported for age-related diseases in humans. A recent study that analysed the same Olink dataset in UK Biobank for associations with the onset of 23 diseases also highlighted GDF15^33^, suggesting that this protein has a widespread impact on human health. GDF15 plays a role in anorexia, lipolysis, and muscle wasting. Furthermore, the sensitivity of some mothers to fetally derived GDF15 has been shown to be a key mechanism for hyperemesis gravidarum^34^ and GDF15 is currently studied as a therapeutic target for the treatment of cancer cachexia^35^. The most important feature according to the SHAP values are the levels of SCARF2 (Supplementary Table 2). SCARF2 is a scavenger receptor protein but remains largely uncharacterised. A recent study identified SCARF2 as a prognostic biomarker for liver cancer in UK Biobank (Supplementary Table 3)^22^. There is some evidence to suggest that SCARF1, a molecule closely related to SCARF2, plays a role in maintaining liver tissue integrity^36^.

Despite both proteomic and methylation-based clocks having a high correlation with chronological age, the degree of shared genetic architecture between these two was modest (proteomic age acceleration vs four epigenetic clocks *R*_*g*_ = 0.17–0.19, all *P* < 0.05). This suggests that that these clocks highlight distinct aging processes. Proteomic age acceleration is also genetically correlated with shorter telomeres, a more narrowly defined marker of cellular aging (*R*_*g*_ = -0.20, *P* = 5×10^–8^). There is some orthogonal evidence for an effect on human aging for all three of our most robustly identified loci. *TET2* encodes an enzyme that catalyses the oxidation of methylcytosine to hydroxymethylcytosine, which is the first step of DNA demethylation^37^. It is a well-characterised CHIP gene^38^ and this had been hypothesised to be the primary mechanism that links *TET2* to aging^39^. However, this hypothesis was not supported by our data. *BRCA1* codes for a protein that plays a key role in the repair of DNA double-strand breaks by homologous recombination^40^. This drives both the well-studied association of rare *BRCA1* variants with breast and ovarian cancer risk^41^ and the relationship between *BRCA1* variation and reproductive aging^42^. However, the best proxy (rs8070085) for our lead proteomic age acceleration SNP at *BRCA1* showed no association with breast cancer risk. It is intriguing that the proteomic age acceleration increasing allele at *BRCA1* also reduced telomere length, a known trigger of cellular senescence^43^. However, it was nominally associated in the opposite direction with IEAA, this highlights the potential complexity of -omics-derived aging phenotypes. *POLR2A* encodes the largest subunit of RNA polymerase II, the polymerase essential for mRNA transcription in eukaryotes. In zones of increased transcriptional activity, POLR2A binds more often at CpG sites causal to aging^44^. If the variant we detect modifies POLR2A function, this could dysregulate transcription, leading to consequences for the proteome^45,46^.

We saw evidence of a causal effect of metabolic health on proteomic age acceleration in our Mendelian randomisation analysis. Both Type 2 diabetes and BMI were strongly associated with faster proteomic aging. BMI has long been known to have a very substantial impact on the proteomic profile of an individual. Sun et al.^47^ found that the majority of proteins in the UK Biobank Olink data had a positive association with BMI in adults. Our results suggest that the specific proteomic dysregulation that accompanies age is also influenced by the features of metabolic syndrome. BMI has also been shown to impact other measures of biological aging with a systematic review linking obesity to accelerated epigenetic aging^9^. The effect of obesity on epigenetic aging has been previously shown^48^, and others have used genetics to highlight more general links between BMI and omics-based aging^10^. These findings support the widespread adverse influence of obesity and related metabolic changes on human aging. The phenotypic analyses reported in Argentieri et al.^16^ suggest that their proteomic age acceleration score is a worse predictor of Type 2 diabetes compared to other age-related diseases (HR for T2D = 1.026 [1.012–1.041] compared to HR for Alzheimer’s disease = 1.157 [1.118–1.975]). In a model adjusting for age, sex and a range of other covariates type 2 diabetes was ranked 13^th^ out of 15 in terms of hazard ratio compared to other clinical outcomes. Taken together with our evidence from the MR analyses this is more consistent with metabolic dysregulation being upstream of proteomic aging rather than downstream of it.

We acknowledge several limitations of our study. Due to the limited current availability of the combination of genetic and proteomic data we were unable to replicate the variant associations directly, though we were able to show genetic correlation with other age-related phenotypes, and some suggestion of association with the variants at *TET2* and *BRCA1* specifically. Proteomic aging is not a single process, and we found discordant disease associations between the three highlighted individual signals and overall protein acceleration. Current data were available only in a small subset of UK Biobank. Larger studies will allow more detailed examination of the distinct processes that contribute to proteomic aging. We assume that trans-pQTL associations are true biological effects and not artefacts related to plasma sample preparation. This has been observed for broadly acting trans-pQTLs in past studies^49^. However, we think that both because of the standardised sample handling and because of the many biologically relevant associations found using the UK Biobank Olink data that these are minor concerns. Another limitation is that our study is specific to individuals of European ancestry, so it is unclear how relevant our findings are to more diverse populations.

In conclusion, we conducted the largest GWAS to-date of proteomic age acceleration. This metric of proteomic age acceleration potentially mediates the relationship between chronological age and major non-communicable diseases. Two of the three signals with widespread effects on plasma protein aging map to *BRCA1* and *TET2*, genes that have been previously linked to human lifespan. Variation in these genes appears to affect methylation and proteomic aging via distinct mechanisms. We find that both increased BMI and the presence of type 2 diabetes have a causal link to faster proteomic age acceleration. Future work should focus on the replication of these results in different cohorts and using different protein assays. Further investigation will also be needed to elucidate the pleiotropic effects of *BRCA1* and *TET2* on aging-related phenotypes.

## Methods

### Calculation of proteomic aging trait

To derive a proteomic aging measure, we started the analysis with 48,108 individuals of broadly European genetic ancestry^50^ from the UK Biobank study for whom proteomic data generated with the Olink Explore 1536 platform was available. Pre-processing and quality control of the UK Biobank Olink proteomics data have been performed by the UK Biobank Pharma Proteomics Project and are described in Sun et al.^47^. The pre-processed data we analysed were provided in the relative NPX unit on a log_2_ scale. We excluded individuals with >10% missingness across all proteins and then excluded proteins with >10% missingness across all individuals which is consistent with the approach proposed by Gadd et al.^33^. Subsequently, we imputed the remaining missing values to the sex-specific means. This resulted in a cleaned dataset of 44,435 individuals typed across 1459 proteins. For the construction of the predicted proteomic aging trait, we employed elastic net regression with 10-fold cross-validation. We set the hyperparameter α to 0.5 reflecting a balance between ridge and lasso regression. The other hyperparameter λ was estimated using 10-fold cross-validation and we found that its optimal value i.e. the one with the smallest root mean square error was 0.001. This reflects that the model performs better with less shrinkage of the coefficients. The elastic net approach was implemented using the R libraries caret (v6.0.94)^51^ and glmnet (v4.1.7)^52^. As we did not apply further feature selection beyond this method, while all available proteins are used in the construction of the model, the majority are weighted such that they are non-informative.

### Identification of most impact proteins using SHAP values and their statistical analysis

We first selected a computationally tractable set of the top 100 proteins by absolute value of their coefficient from the elastic net. To further refine this feature set, we calculated SHAP (SHapley Additive exPlanations)^18^ values for each protein. These values quantify the contribution of an individual feature to the model’s predictions. We then ranked the proteins by their average absolute SHAP values and selected the top 20 with the highest contributions. The SHAP value calculations were implemented using the R library fastshap (v0.1.0) (https://github.com/bgreenwell/fastshap)^53^. Cancer associations in UK Biobank for these top 20 proteins were retrieved from the supplementary materials provided by Papier et al.^22^ for their comprehensive analysis of cancer-protein associations.

### Phenotypic derivation and associations

We used regression models to assess the relationship between a selected group of age-related phenotypes and diseases. We use two cancer variables, one based on any diagnosis of cancer, and one excluding those who had a diagnosis of blood cancer only^54^. Diabetes was defined on a report of either incidence or prevalent type 2 diabetes^50^. Ischemic Heart Disease was coded as a binary variable as follows: an individual was considered a case if they had any of the ICD-10 codes I20-I25. This information was extracted using the “first occurrence” data available generated by the UK Biobank team using fields 131296, 131298, 131300, 131302, 131304 and 131306^55^. The continuous outcomes - loss of the Y chromosome^32^ and age at natural menopause^54^ - have been previously described.

Mortality data was accessed with the UK Biobank RAP on 29/09/2023 with a censoring date of 30/11/2022 for all participants. To assess the impact of proteomic age on survival, we fitted a Cox proportional-hazards model using the R library survival (v3.5.5)^56,57^. We adjusted for age at recruitment and sex. For all variables in the Cox model, we conducted an examination of Schoenfeld residuals^58^. While the assumption of proportionality of hazards was violated for proteomic age, the local hazard ratio was consistently significantly different from the null (Supplementary Fig. 1). The Kaplan-Meier survival plot was implemented with the R library survminer (v0.4.9)^59^.

### Genome-wide association study

We ran the first stage genome-wide association using UK Biobank data in a white European set resulting in an effective sample size of 44,351 individuals, using BOLT-LMM^60^. To identify independent GWAS signals and prioritise causal gene candidates at the resulting loci, we used the “GWAS to genes” pipeline as described in Kentistou et al.^61^ and discussed in brief below. GWAS summary statistics were filtered to retain variants with a MAF > 0.1%. Quasi-independent genome-wide significant (GWS) signals were initially selected in 1 Mb windows and secondary signals within these loci were further selected via conditional analysis in GCTA^62^, using an LD reference derived from the UK Biobank study. Primary signals were then supplemented with unlinked (R^2^ < 5%) secondary signals, whose association statistics did not overtly change in the conditional models and signals were mapped to proximal NCBI RefSeq genes, within 500 kb windows.

Independent signals and closely linked SNPs (R^2^ > 0.8) within the associated loci were annotated if they were coding variants within the identified genes or if they mapped within known enhancers of the identified genes, via the ABC enhancer maps^63^. Signals were first annotated with their physically closest gene. Gene-level associations were then determined via MAGMA^64^, by collapsing all coding variants within a gene. Colocalization analyses between the GWAS and eQTL or pQTL^47,65^ data were also performed via SMR HEIDI (v0.68)^66^ and the ABF function within the R package “coloc” (v5.1.0)^67^. For eQTL analyses, these were applied for specifically enriched tissues (via LDSC-SEG)^68^, as well as cross-tissue meta-analysed GTEx eQTL data^69^ and data from the eQTLGen^70^ and Brain-eMeta^71^ studies. Finally, genes within the associated loci were also prioritised through the PoPS^72^ method. Causal candidate genes were then prioritised by overlaying all of the above information and scoring the strength of evidence observed. For further details about the specific application of this method, see Kentistou et al.^61^. As discussed in the results, at one site we found that there are two very highly ranked genes with very equitable scores. In this instance, we made a choice based on the biological literature.

We assess if our top hits were pQTLs for any of the proteins available in UK Biobank using a linear regression model, with a *P*-value cut-off of (0.05/1463 = 3.4×10^–5^). To adjudge the possibility of our top hits only being associated with a protein very highly weighted in the model, we made a new locus-specific elastic net for each of our regions removing any of the proteins associated with the signal in the generation of the model. Once this new elastic-net was generated we re-ran the GWAS.

### LDSC

We used linkage disequilibrium score regression (LDSC)^73^ to compute the pairwise genetic correlations between proteomic age acceleration and other established biological age scores or biomarkers including four epigenetic clocks: Hannum^3^, IEAA^25^, DNAm PhenoAge^26^ and GrimAge^27^ and telomere length. We utilised the default LD score reference panel provided by LDSC. The summary statistics of four epigenetic clocks and telomere length were extracted from McCartney et al.^10^ and Codd et al.^74^, respectively.

### Exome burden tests

#### UK biobank data processing and quality control

We used the same processing strategies as outlined in our previous paper to analyse the whole-exome sequencing data and perform quality control steps^50,75^. We queried whole-exome sequencing data from 469,835 individuals in the UK Biobank^76^, excluding those with excess heterozygosity, autosomal variant missingness on genotyping arrays >=5%, or those not included in the subset of phased samples as defined by Bycroft et al.^77^.

The whole-exome sequencing data was stored as population-level VCF files, aligned to GRCh38, and accessed through the UK Biobank RAP. In addition to the quality control measures already applied to the released data, which were described by Backman et al.^76^ we conducted several extra QC procedures. Firstly, we used ‘bcftools norm’^78^ to split the multiallelic sites and left-correct and normalise InDels. Next, we filtered out variants that failed our QC criteria, including: 1) read depth < 7, 2) genotype quality < 20, and 3) binomial test *p*-value for alternate allele reads versus reference allele reads <=0.001 for heterozygous genotypes. For InDel genotypes, we only kept variants with read depth >=10 and genotype quality >=20. Variants that failed QC criteria were marked as missing (i.e,./.). After filtering, variants where more than 50% of genotypes were missing were excluded from downstream analyses^50^.

The remaining variants underwent annotation using ENSEMBL Variant Effect Predictor (VEP) v108^79^ with the ‘-everything’ flag, and additional plugins for REVEL^80^, CADD^81^, and LOFTEE^82^ For each variant, a single ENSEMBL transcript was prioritised based on whether the annotated transcript was protein-coding, MANE select v1.0^83^ or the VEP Canonical transcript. The individual consequence for each variant was then prioritised based on severity as defined by VEP. Stop-gained, splice acceptor, and splice donor variants were merged into a combined Protein-Truncating Variant category, while annotations for missense and synonymous variants were adopted directly from VEP. We only included variants on autosomes and the X chromosome that were within ENSEMBL protein-coding transcripts and within transcripts included on the UK Biobank whole exome sequencing (WES) assay in our downstream analysis. Our analyses focused primarily on individuals of European genetic ancestry, and we excluded those who withdrew consent from the study.

#### Exome-wide gene burden testing in the UK Biobank

We used BOLT-LMM v2.3.6^60^ as our primary analytical tool to conduct the gene burden test. To run BOLT-LMM, we first queried a set of genotypes with MAC > 100 which derived from the genotyping arrays for the individuals with the WES data to build the null model. To accommodate BOLT-LMM’s requirement for imputed genotyping data rather than per-gene carrier status, we developed dummy genotype files where each gene was represented by a single variant. We then coded individuals with a qualifying variant within a gene as heterozygous, regardless of the total number of variants they carried in that gene. We then created the dummy genotypes for the MAF < 0.1% high confidence PTVs as defined by LOFTEE, missense variants with REVEL > 0.5 and missense variants with REVEL > 0.7. We then used BOLT-LMM to analyse phenotypes, using default parameters except for the inclusion of the ‘lmmInfOnly’ flag. In addition to the dummy genotypes, we also included all individual markers contained in WES data to generate the association test statistics for individual variants. We used the number of proteins measured for each sample, assessment centres, sex, the first 20 genetic principal components as calculated by Bycroft et al.^77^ and the WES release batch (50k, 200k, 450k, 470k) as covariates.

#### Common variant GWAS lookups

Loci were looked up in FinnGen^84^ via https://r9.finngen.fi/ (accessed on 13/10/2023), GWAS Catalog^85^ via https://www.ebi.ac.uk/gwas/ (accessed on 03/10/2023) and the Open Targets Genetics platform release *22.10*^86^ via https://genetics.opentargets.org/ (accessed on 22/09/2023).

#### MR

We ran Mendelian randomisation models to assess the possible causal influence of a number of age and health-related phenotypes on proteomic age acceleration. We assessed the impact of height^87^, type 2 diabetes^88^ BMI^89^, loss of the Y chromosome^32^ and age at menopause^28^. We also looked at the impact of sex hormone levels^31^, in these cases we considered the impact on a sex-specific model of proteomic aging. In each case we ran IVW (Inverse Variance Weighted) and MR-Egger models to identify any associations.

## Supplementary information

Supplementary Information

Supplementary Tables

## Data Availability

The UK Biobank phenotype, proteomics and whole-exome sequencing data described here are publicly available to registered researchers through the UK Biobank data access protocol. Information about registration for access to the data is available at: https://www.ukbiobank.ac.uk/enable-your-research/apply-for-access. Data for this study were obtained under Resource Application 9905. Other publicly available datasets we used: Epigenetic age acceleration GWAS summary statistics: https://datashare.ed.ac.uk/handle/10283/3645. Telomere length GWAS summary statistics: https://figshare.com/s/caa99dc0f76d62990195. FinnGen data freeze 9: https://r9.finngen.fi/ GWAS Catalog: https://www.ebi.ac.uk/gwas/. Open Targets Genetics platform release 22.1081: https://genetics.opentargets.org/. Summary statistics from the GWAS of proteomic age acceleration are available via 10.17863/CAM.116389.

## Code availability

Elastic net regression was implemented using the R libraries caret (v6.0.94) (https://github.com/topepo/caret) and glmnet (v4.1.7). (https://github.com/cran/glmnet). The SHAP value calculations were implemented using the R library fastshap (v0.1.0) (https://github.com/bgreenwell/fastshap). Survival analyses were performed using the R libraries survival (v3.5.5) (https://github.com/therneau/survival) and survminer (v0.4.9) (https://github.com/kassambara/survminer). Genome-wide association analyses on SNP data were run using BOLT-LMM (v2.3.2) (https://alkesgroup.broadinstitute.org/BOLT-LMM/downloads/old/). Conditionally independent signals were identified using GCTA (v1.92.0). LDSC-SEG (https://github.com/bulik/ldsc, v1.0.1) was used to perform tissue enrichment analyses. eQTL and pQTL colocalization analyses were performed using the R package ‘coloc’ (v5.1.087) and SMR-HEIDI (v0.6886). We used MAGMA (v1.09) to determine gene-level associations and PoPS (https://github.com/FinucaneLab/pops, v0.2) to prioritise genes within associated loci. We computed pairwise genetic correlations using LDSC (https://github.com/bulik/ldsc, v1.0.1). Code for WES data processing and association testing is available on GitHub (https://github.com/mrcepid-rap/mrcepid-runassociationtesting). Plots were created using R (v4.2.2) using ggplot2 (v3.4.2).
