# Supplementary material for: Genetic determinants of proteomic aging
Source: NPJ Aging. 2025 Apr 26;11(1):30. doi: 10.1038/s41514-025-00205-4 (PMC12033249; doi:10.1038/s41514-025-00205-4)
Supplement: Supplementary file 1 — Supplementary Information [file 41514_2025_205_MOESM1_ESM.pdf]

Supplementary Information

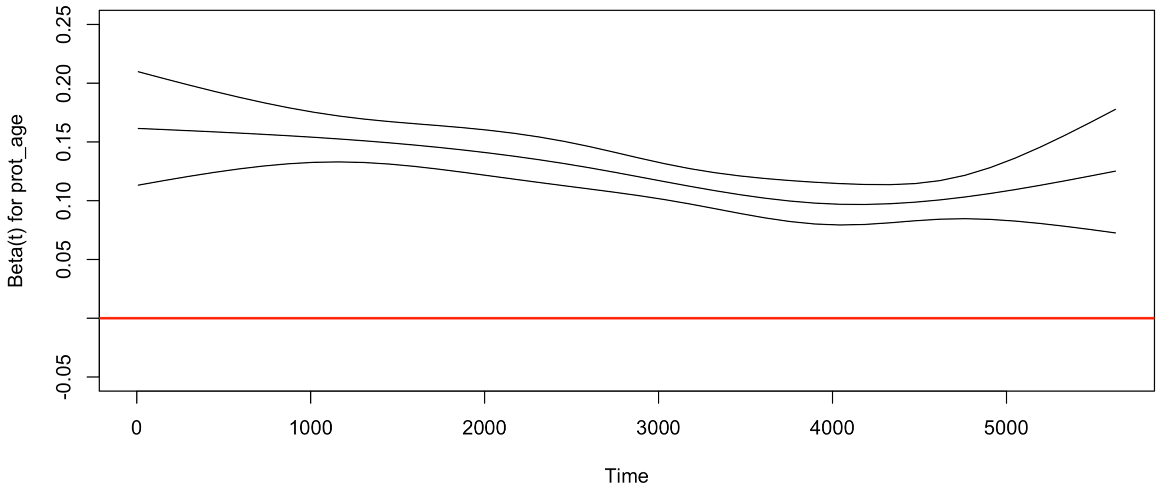

Supplementary Figure 1: Plot of hazard ratio estimated for proteomic age as predictor of mortality. Note that time is given in days.

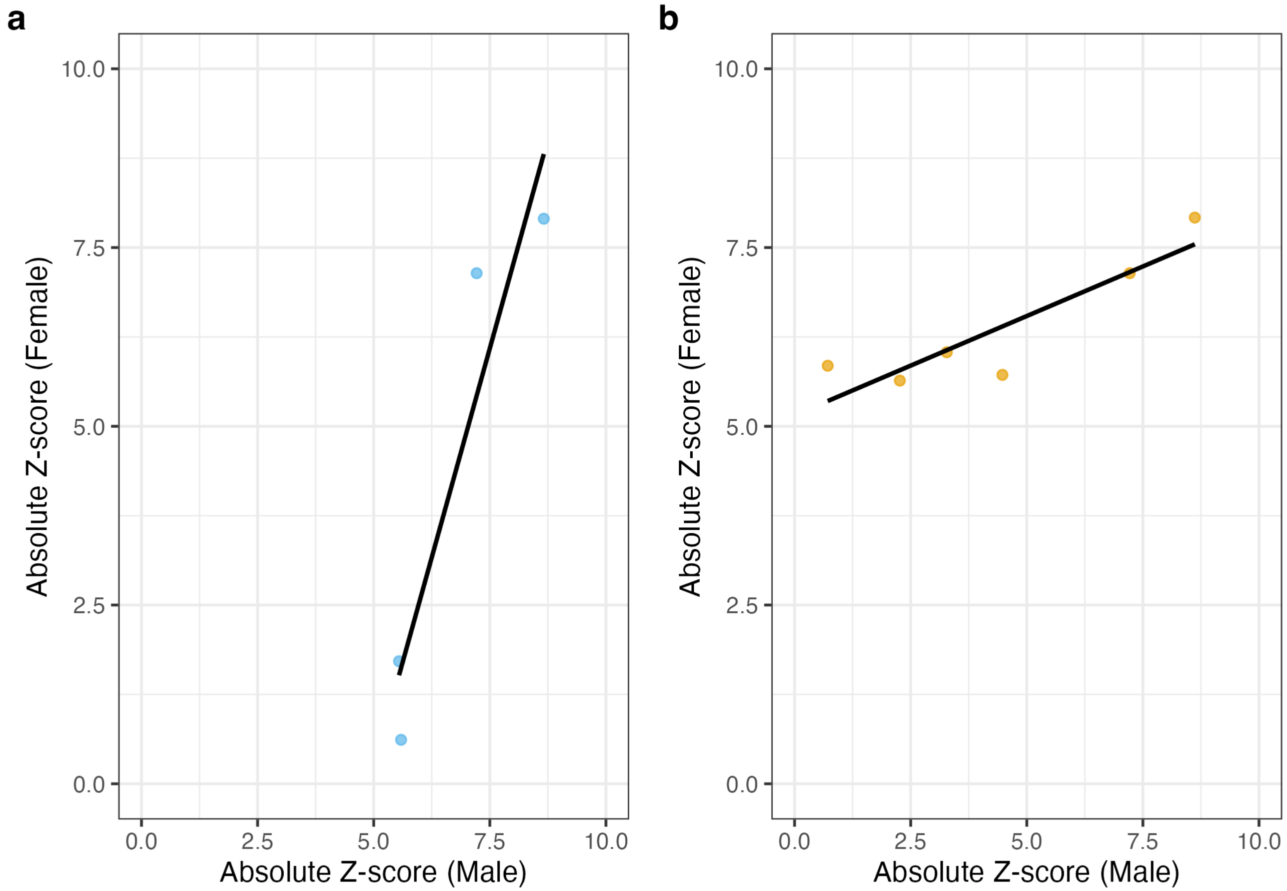

Supplementary Figure 2: Sex-specification replication analyses. In both analyses, significant loci from the sex-specific GWAS were identified using distance-based clumping within 1 Mb windows. a) scatter plot for z-score effect sizes for 4 variants that reached genome-wide significance for analysis of proteomic aging in men only. b) scatter plot for z-score effect sizes for 6 variants that reached genome-wide significance for analysis of proteomic aging in women only. Spearman's correlation coefficient ρ was 0.80 for variants identified from male-only analysis and 0.71 for variants identified from female-only analysis.

Supplementary Note

To identify rare variants associated with proteomic age acceleration, we performed exome-wide gene-burden tests using data from 42,735 individuals of European ancestry, whose whole exome sequencing and proteomics data are available in UK Biobank (**Supplementary Table 9**). Individual gene-burden tests were performed by collapsing rare (MAF<0.1%) genetic variants for each gene. We tested three categories of variants based on their predicted functional impact: high-confidence Protein Truncating Variants (PTVs), and two overlapping missense masks that used a REVEL score[1] threshold of 0.5 or 0.7. This yielded a total of 21,935 gene tests with at least 15 informative rare allele carriers, corresponding to a multiple-test corrected statistical significance threshold of $P<2.28\times10^{-6}$ (0.05/21,935). We then used BOLT-LMM to perform the association testing. No genes passed the statistical significance threshold in our analysis. The most significant gene burden identified was the missense variants with REVEL score >0.7 in *THBS4* (Thrombospondin 4, beta=0.92, SE=0.2, $P=5.2\times10^{-6}$, carrier N=198). Gene-burden tests for the three GWAS genes with widespread effects on plasma proteomic aging (*TET2, BRCA1* and *POLR2A)* showed a nominal significant association between proteomic age acceleration and PTVs in *TET2* (beta=-1.6, SE=0.5, $P=5\times10^{-4}$, carrier N=36). As *TET2* is a well-known clonal haematopoiesis of indeterminate potential (CHIP) gene, whose somatic mutations are strongly associated with age, we then extracted the possible CHIP mutations (MAF≤0.35, N=15) in *TET2* and ran a burden test for the possible CHIP mutations. We did not see a significant association (beta=-0.64, SE=0.7, *P*=0.4), and after removing these variants, the association between PTVs in *TET2* became more significant (beta=-2.5, SE=0.6, $P=5\times10^{-5}$). The associations of *BRCA1* and *POLR2A* with proteomic age acceleration were not significant (for *BRCA1* PTVs: beta=0.28, SE=0.46, P=0.55, carrier N=38; for *POLR2A* PTVs: beta=0.02, SE=0.58, P=0.98, carrier N=24).

References

1. Ioannidis, N. M. *et al.* REVEL: An Ensemble Method for Predicting the Pathogenicity of Rare Missense Variants. *Am J Hum Genet* **99**, 877–885 (2016).
